# Public discourses of alternative protein foods in Facebook public pages’ posts, 2014–2024

**DOI:** 10.1371/journal.pone.0333922

**Published:** 2025-10-17

**Authors:** Weiyu Zhang, Bingyan Wang, Jingyi Xie, Lai Peng Leong, Tai Wei David Leong, Max Jing Rui Tham, Wee Han Ang

**Affiliations:** 1 Department of Communications and New Media, National University of Singapore, Singapore, Singapore; 2 Department of Political Science, Tsinghua University, Beijing, China; 3 Department of Food Science and Technology, National University of Singapore, Singapore, Singapore; 4 Department of Chemical and Biomolecular Engineering, National University of Singapore, Singapore, Singapore; 5 Department of Chemistry, National University of Singapore, Singapore, Singapore; Philadelphia University, JORDAN

## Abstract

“Alternative protein” (AP) refers to protein sources that are different from conventional animal-based proteins. This category encompasses a variety of highly processed plant-based proteins, cultured or cell-based meat, and is primarily driven by factors like sustainability, health, ethical concerns about animal welfare, and the need to feed a growing global population. The aim of this study is to map and understand public discourses around AP foods through social monitoring, in particular, computational analyses of social media big data. Through analyzing the topics and sentiments of Facebook public pages’ English content in the decade of 2014–2024, we found that (1) public attention on plant-based AP foods dropped since 2021, while attention on cell-based AP foods showed an uneven growth; (2) public sentiments towards plant-based AP foods were consistently positive, while sentiments towards cell-based AP foods were clearly more mixed; (3) discussion topics under plant-based AP foods were more practical and diverse than topics under cell-based AP foods; (4) the leading actors talking about AP foods were enterprises & industry, media, and organizations. Overall, plant-based AP foods have generated a more established and diverse public discourse, while cell-based AP foods are still emerging in public conversations and continue to face greater barriers to widespread understanding and acceptance.

## Introduction

“Alternative protein” (AP) refers to protein sources that are different from conventional animal-based proteins. The motivation behind AP development is mainly from considerations like sustainability [1–3], health [4,5], ethical concerns about animal welfare, and the need to feed a growing global population [5]. AP foods offer potential environmental benefits, such as lower greenhouse gas emissions, reduced land and water usage, and less reliance on animal farming, making them a key focus in discussions about sustainable food systems and the future of food security. Types of alternative proteins include plant-based proteins, cultured or cell-based meat [6,7], insect protein, mycoprotein derived from fungi, algae and seaweed protein [8], and fermented proteins [9]. Among the various types, plant-based and cell-based alternative proteins have captured the highest interests from both the scientific community and the publics [10,11].

The commercialization of alternative proteins has progressed in tandem with technological innovations, underpinned by shifting consumer preferences and increased investment in sustainable food systems. Since the first vegetarian burger made by *Quorn* in 1985, companies like *Beyond Meat* and *Impossible Foods* have played pivotal roles in transforming consumer perceptions by launching products with enhanced taste, texture, and nutritional profiles [9,12]. Cell-based meat, although technologically promising, faces commercialization challenges due to high production costs and regulatory hurdles. Despite these barriers, the industry has made strides with significant investments in R&D and strategic partnerships. Countries like Singapore have pioneered regulatory frameworks, approving cell-based chicken for sale, signaling the potential for broader acceptance [7]. The alternative protein industry has also benefited from robust funding from venture capital and governmental initiatives aimed at fostering innovation [13]. The global market for alternative proteins, estimated at USD 14 billion, is set for exponential growth [14].

Despite of the scientific and commercial breakthroughs in developing alternative protein foods, public understanding and acceptance of alternative protein foods have been growing but rather slowly. A study [15] that tracked consumer acceptance of insect protein in Hungary between 2016 and 2021 found that 70% of Hungarians did not want to try entomophagy in both years. In Belgium, although consumers who said existing plant-based meat alternatives met their needs increased significantly from 44% in 2019 to 51% in 2020, their attitudes towards cultured meat between the two waves did not change [16]. A review article [17] found studies that demonstrate that in 2013, 19% of UK adults would eat “artificial meat” and in 2017, 16% would eat “cultured meat” grown in a lab.

Most current studies attributed the slow public acceptance of AP foods, despite of active institutional promotions, to consumer perceptions. Consumer concerns over alternative protein foods are multi-faceted. Neophobia of both the foods and the technologies behind them, the disgust factor, and the cultural factors were found to be major barriers in the early adoption of AP foods [17,18]. Although there are well-documented benefits of consuming AP foods, the impacts of such foods on long-term health are still unknown due to the novelty of such food technologies [19]. Price, taste, and consumption venues were also suggested to have influences on consumer decisions [20–22].

Our paper takes a broadened approach to go beyond consumers and see the people involved in AP foods as publics [23]. While consumers make decisions to purchase or not to purchase, publics talk about AP foods and exchange their views. Narratives generated from public debates could be influential in shaping the path of AP [24,25]. Our approach emphasizes the potential impacts of the public, a collective of individuals who communicate with each other to shape and contest our vision of AP foods. This paper aims to understand public discourses around AP foods through social monitoring [26] or analyzing social media data [27]. Social media such as Facebook and Twitter are believed by both experts [28] and ordinary users [29,30] to be important information sources, which can be used to provide both useful [31] and distorted information [32]. Facebook is the most popular social media in the world, with nearly 40% of the world’s population using it [33]. As of Q4 2023, Facebook had 3.065 billion monthly active users. Our paper took advantage of CrowdTangle, a data platform provided by Meta (Facebook’s parent company), before the platform was closed down in 2024. Specifically, this study seeks to systematically map the evolving public discourses around alternative protein foods by examining the topics, sentiments, and key actors involved in English-language Facebook posts over the past decade (2014–2024).

## Materials and methods

This study has been reviewed and approved by National University of Singapore IRB. No written or verbal consent was obtained from the individual Facebook public page owners. Such consent was waived for our study because our aggregative data were obtained through CrowdTangle, Facebook’s official data platform. Instead, our IRB approved our study based on the condition that we follow CrowdTangle’s rules and restrictions. For this reason, our data cannot be made publicly available.

**Fig 1:**
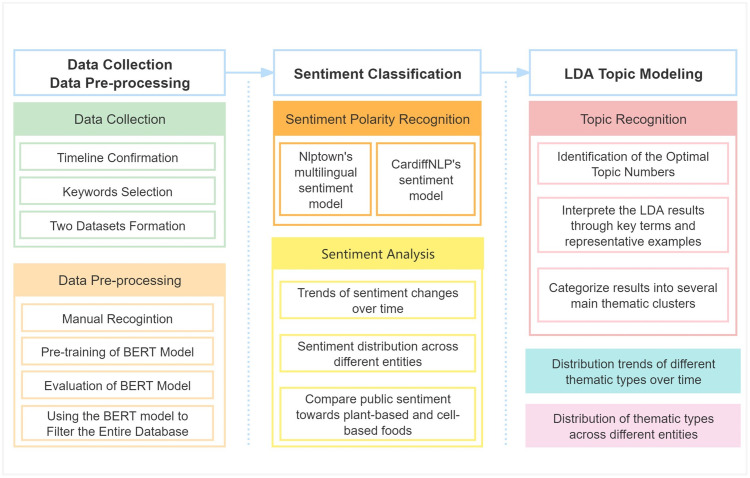
The flowchart of this Research. This figure shows the details of the research process.

We adopt a multi-stage research approach to examine public discourses surrounding alternative proteins, combining data collection, pre-processing, sentiment analysis, and topic modeling. Fig 1 outlines the complete research framework and its key components. The following sections elaborate on each step in detail.

### Data collection

Our data come from Facebook pages archived by CrowdTangle, the official Facebook data platform that was closed in 2024. The collection and analysis method complied with the terms and conditions for the source of the data. According to Facebook, “pages are places on Facebook where artists, public figures, businesses, brands, organizations and non-profits can connect with their fans or customers.” In contrast, “groups are a place to communicate about shared interests with certain people.” Facebook pages are often seen as broadcasting channels that send messages out to their followers while Facebook groups are deemed as communities that emphasize common interests and interactions among group members. One study that examines public pages vs. groups related to vaccines concludes that “public pages may work more efficiently to have targeted vaccine campaigns for organizations or institutions than setting up public group discussions.” [34] When using the CrowdTangle API to search through its database, we included all post types in English Language, regardless whether the post was branded content or not.

We set the search timeframe from 19 July 2014–18 July 2024, spanning over ten years. The data was accessed on 18 July 2024. Individual participants cannot be identified as the data are from public pages, not private users. The one-decade timeframe is also the longest timeframe CrowdTangle allows researchers to set. Our search keywords have two broad topic focuses: plant-based vs. cell-based protein foods. Other than including as many relevant word combinations as we can imagine, we used a typical Boolean technique to exclude applications of plant-based or cell-based technologies to produce non-food products such as diamond or to address other non-food challenges such as curing cancers. Details of our search keywords set-up can be found in Supporting Information S1 Table. The first-round search yielded 288,297 posts for plant-based and 48,681 for cell-based datasets.

### Data pre-processing

For both plant-based and cell-based datasets, two human coders independently pre-coded a sample of 200 Facebook posts. The inter-coder reliability, assessed using Krippendorff’s alpha (α), were satisfactory (0.966 for plant-based and 0.879 for cell-based datasets). A set of coding criteria was established (details can be found in Supporting Information S1 Appendix) and the two coders performed an additional coding of 800 posts. These 1000 posts served as the training set for later computational categorization of data. We used BERT [35] model to classify the rest of posts as either relevant or irrelevant to our search topics. We firstly partitioned the dataset, allocating 80% for training and reserving the remaining 20% for validation. The training dataset was further divided into training and test subsets. We selected the model that demonstrated the best performance on the test dataset. The performance metrics for the chosen models are summarized in Supporting Information S1 Appendix. To evaluate the agreement between the predictions generated by the BERT model and those provided by human coders, we further analyzed the validation dataset. The Cohen’s Kappa values indicate the consistency between model-generated vs. human-generated coding and were found to be satisfactory (0.759 for plant-based and 0.828 for cell-based datasets). After this round of relevance cleaning, our datasets were further cleaned to contain 243,775 posts for plant-based and 39,883 for cell-based datasets.

### Sentiment classification analysis

We applied an unsupervised machine learning approach to perform sentiment classification of social media texts. Due to the presence of a significant amount of non-English languages in the posts, we ultimately chose to utilize two mature models, Nlptown and CardiffNLP, for sentiment polarity classification. The model “nlptown/bert-base-multilingual-uncased-sentiment” is a sentiment analysis model based on BERT (Bidirectional Encoder Representations from Transformers), specifically the bert-base-multilingual-uncased version. This version of BERT was pretrained on a large multilingual corpus covering over 100 languages and fine-tuned for sentiment classification tasks. This model has been widely used in industry and academic projects [36–38]. The model ultimately outputs a five-point scale, ranging from “very negative” to “very positive,” which provides a nuanced representation of sentiment and captures subtle emotional gradients within the text. To standardize the classification output, we classify scores 1–2 as “negative,” 3 as “neutral,” and 4–5 as “positive.” The “cardiffnlp/twitter-xlm-roberta-base-sentiment” model is a state-of-the-art multilingual sentiment analysis tool fine-tuned on Twitter data. It is built upon XLM-RoBERTa, a transformer-based multilingual model developed using the robust RoBERTa architecture, which is optimized for performance on cross-lingual natural language processing tasks. The model is trained to classify texts into three sentiment categories: positive, neutral, and negative. Its fine-tuning on Twitter data provides a significant advantage in capturing the nuances of social media texts, including informal expressions, abbreviations, and platform-specific linguistic patterns.

In this study, we employed the two well-established sentiment analysis models to analyze social media posts related to plant-based and cell-based foods. All posts were batch-processed and assigned sentiment labels according to the classification scheme outlined above. The latter model serves as a robustness check for the former, leveraging its fine-tuning on social media-specific language to enhance reliability in this context. By integrating the results from both models, we aim to derive a comprehensive understanding of public sentiment toward AP foods. We visualized the sentiment proportions over time and conducted descriptive comparisons across categories using bar charts and line graphs. This approach not only provides valuable insights into the emotional and attitudinal nuances expressed in online discourses but also enables us to analyze the temporal trends in public sentiment over time. In addition, we examine the variations in sentiment distribution across different entities (i.e., page categories), shedding light on how different page categories or actors engage with these topics. Furthermore, we compare the emotional tones in posts about plant-based vs. cell-based foods, identifying any significant differences in public discourses regarding these two emerging food categories.

### LDA topic modeling

We used an unsupervised machine learning method, Latent Dirichlet Allocation (LDA), to identify latent topics from social media texts. Latent Dirichlet Allocation (LDA) is a widely used unsupervised topic modeling technique [39], assuming that each document is a mixture of a small number of latent topics and that each word in the document is attributable to one of those topics [40,41]. LDA has become a standard method in the field of text mining due to its simplicity, interpretability, and ability to uncover hidden thematic structures in large text corpora. Given that the databases include cross-lingual data, we first addressed the challenge of non-English texts. While LDA can effectively model multilingual data, the presence of non-English texts can complicate the interpretation and subsequent analysis. Therefore, we utilized the “textcat” package in R to filter out non-English texts. After this pre-processing step, the plant-based foods database retained 189,403 records, and the cell-based foods database contained 33,989 records.

Subsequently, we employed the “stm” (Structural Topic Model) package in R to perform topic modeling separately on both databases. To determine the optimal number of topics for each corpus, we evaluated the model results based on four criteria: exclusivity, semantic coherence, held-out likelihood, and residuals. Semantic coherence measures the degree to which top words in a topic co-occur, while exclusivity reflects the uniqueness of topic words. Held-out likelihood evaluates model predictive performance, and residuals indicate unexplained variance in the model. These indicators allow us to balance the clarity and interpretability of the topics with the model’s overall fit. Following the topic modeling, we analyzed the key terms and representative examples provided by the LDA results to interpret the topics. Based on these insights, we consolidated the topics into several main thematic clusters. Finally, we examined the distribution trends of different topics over time and the distribution of topics across different entities for both plant-based and cell-based foods datasets. A comparative analysis was then conducted to identify similarities and differences between the two datasets, providing a deeper understanding of how thematic discourse evolve across the two categories of AP foods.

## Results and discussion

### Public attention to AP foods over the past decade

**Fig 2:**
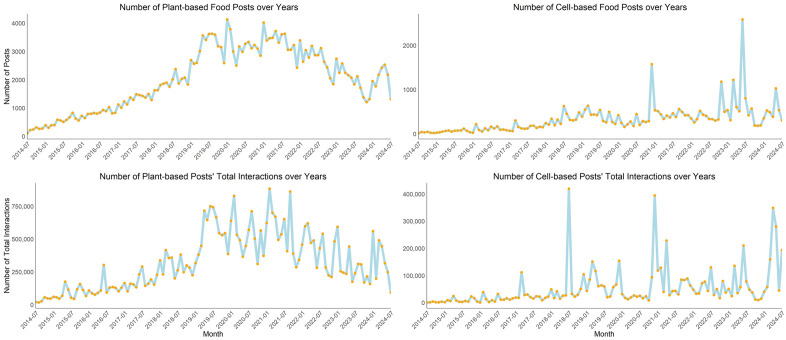
Number of Plant-based vs. Cell-based Foods Posts and Total Interactions over Years.

First, we summarize global trends in public attention to plant-based vs. cell-based AP foods over the past decade. Due to the database not including a complete set of posts for 2014 and 2024, data from the two years were excluded from the trend analysis. As shown in Fig 2, the number of posts related to plant-based and cell-based foods experienced rapid growth from 2015 to 2019, whereas this growth slowed down significantly after 2019. Posts about plant-based foods began to decline notably after 2021, returning to 2018 levels by 2023, while posts about cell-based foods saw a resurgence after 2022, reaching a decade-high peak. A similar pattern is observed in total interactions with posts, with the only difference that after 2022 interactions with plant-based foods posts decreased significantly, while those with cell-based foods posts increased markedly. In brief, over the past decade, public interest in AP foods has fluctuated, moving from rapid growth to a period of cooling down. After 2022, public attention to plant-based vs. cell-based foods began to diverge: interest in plant-based foods continued to decline, whereas attention to cell-based foods saw a notable resurgence. This trend analysis is cross-checked using Google Trends (see Supporting Information S1 Fig for more details) and the findings are consistent.

We took a closer look at the peaks in the trend analysis and found that they correspond to prominent regulatory and industry breakthroughs. For plant-based foods, the highest peak in number of posts appeared in January 2020. At the Consumer Electronics Show (CES) 2020 in Las Vegas, *Impossible Foods* launched its Impossible Pork and Impossible Sausage products, the company’s first foray beyond beef alternatives. For plant-based foods, the highest peak in number of interaction occurred around early 2021. In February 2021, *Beyond Meat* entered into partnerships with *McDonald’s* and *Yum! Brands*. These collaborations aimed to introduce plant-based options across various menus, including the “McPlant” burger for McDonald’s and plant-based pizza toppings, chicken alternatives, and potential taco fillings for Yum! Brands’ restaurants such as *KFC*, *Taco Bell*, and *Pizza Hut*. For cell-based foods, a clear peak appeared in June 2023 when the U.S. Department of Agriculture (USDA) granted approval for the sale of cell-cultivated chicken products, allowing companies like *Upside Foods* and *Good Meat* to introduce their products to the U.S. market. For cell-based foods, the highest peak in number of interaction occurred as early as around July 2018. In that month, USDA and the Food and Drug Administration (FDA) began formal discussions to determine how cell-based meats should be regulated.

**Fig 3:**
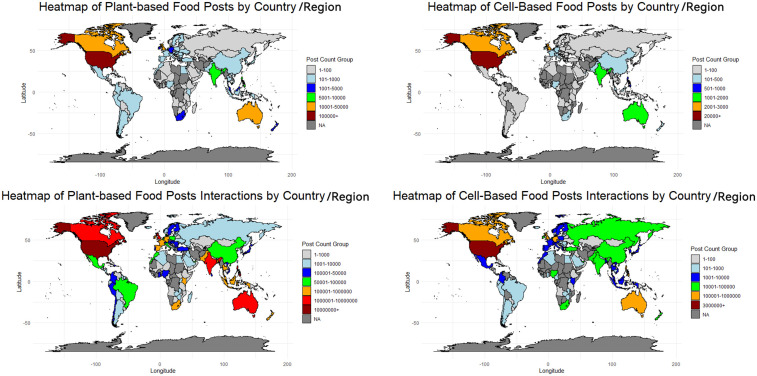
Heatmap of AP Food Posts and Total Interactions by Country/Region.

We then examined the level of public attention to AP foods across different countries/regions. Fig 3 presents a heatmap illustrating the distribution of posts on AP foods by country/region, along with their total interactions. According to the figure, a highly uneven global distribution of both posts and interactions surrounding AP foods can be detected obviously. Regarding plant-based AP foods, the United States demonstrates the highest level of public interest, followed by the United Kingdom, Australia, and Canada. Other countries, such as India, the Philippines, New Zealand, Germany, and South Africa, show a third tier of engagement. The global distribution of posts on cell-based AP foods follows a similar pattern to that of plant-based foods. The United States leads by a considerable margin, followed by the United Kingdom and Canada. Other countries, including Australia, India, and the Philippines, fall into subsequent tiers. The distribution of total interactions with posts on AP foods mirrors that of the number of posts, with some slight differences. For instance, Malaysia, Thailand, and France rank relatively higher in total interactions compared to post volume in discussions on plant-based foods. Countries like India, South Africa, China, and Russia demonstrate a higher ranking in total interactions than post volume when it comes to discussions on cell-based foods.

We continued to investigate which page categories or actors are more interested in the topic of AP foods, as evidenced by the number of related posts they publish. Based on the page category information provided by Facebook, pages are classified into 929 categories. After human consolidation and classification, we divided them into six major page categories: Enterprise & Industry, Government, Media, Organization, Person, and Technology (see Supporting Information S2 Appendix for more details). For plant-based AP foods, the Enterprise & Industry group shows the highest number of posts, followed by Organization and Media in second and third place, respectively. For cell-based AP foods, Media publish the highest number of posts, with Organization and Enterprise & Industry ranking second and third. In short, while the page categories interested in AP foods are becoming more diverse, the majority of related discourses still come from the media, organizations, and enterprises/industries (see Supporting Information S2 Fig for more details).

### Public sentiments towards AP foods

**Fig 4:**
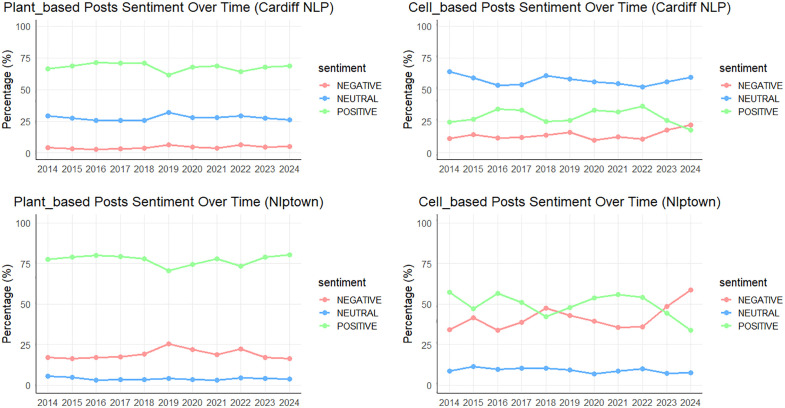
Sentiment of Plant-based and Cell-based Foods Posts over Years.

Fig 4 illustrates the trend of sentiment polarity for plant-based vs. cell-based AP foods posts over the years. We used two models to test (the Nlptown model and CardiffNLP model) and both demonstrate that approximately 70% of social media posts discussing plant-based foods express positive sentiment, with a relatively smaller proportion reflecting neutral or negative emotions. It is evident that the proportions of negative and neutral emotions in cell-based foods posts significantly exceed those in plant-based foods posts. According to the results of cell-based foods posts from the Nlptown model, the proportions of negative and positive sentiments are generally comparable, fluctuating from 30% to 50% each. Besides, in the years 2018, 2023, and 2024, the proportion of negative sentiment surpasses that of positive sentiment, with notable fluctuations in sentiment proportions across these years. In contrast, the CardiffNLP model reveals that neutral sentiment holds the highest proportion in cell-based posts, while the proportion of positive sentiment shows a notable decrease compared to plant-based posts, and the proportion of negative sentiment exhibits a slight increase. In summary, the results of the sentiment analysis were very similar to our initial expectation that the publics have more negative attitudes toward cell-based AP foods.

**Fig 5:**
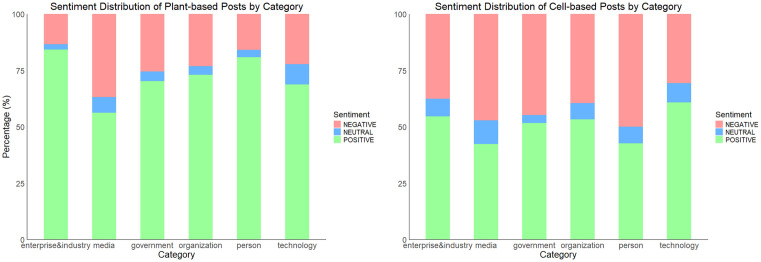
Sentiment Distribution of AP Foods Posts by Page Categories (model: Nlptown).

In addition, our analysis examined the distribution of sentiment across different page categories (see Fig 5). Our findings reveal that ordinary people exhibited a higher proportion of negative sentiment toward cell-based foods compared to other page categories, aligning with previous interpretations that the average consumer is particularly more cautious or critical towards cell-based AP foods.

### Topics in public discourses on AP foods

Our study further applies the Latent Dirichlet Allocation (LDA) model to extract the topics regarding plant-based vs. cell-based AP foods shown from Facebook posts, comparing the similarities and differences between the two. For the analysis of social media posts related to plant-based AP foods, we determined that 11 topics were the optimal number based on a comprehensive evaluation of model performance metrics. Specifically, we used several metrics, including exclusivity, semantic coherence, held-out likelihood, and residuals. Topic number 11 performed relatively well across these metrics, making it the best choice overall. Based on representative keywords from each topic, the 11 topics were further consolidated into five broader types: ***Menus and Recipes****,* ***Products Types****,* ***Health and Nutrition****,* ***Ethical Concerns*** and ***Business Dynamics***. Similarly, we applied the same LDA method to perform topic modeling on cell-based foods posts and the optimal number of topics was determined to be 10. By analyzing the keywords and representative examples for each topic, we grouped them into five major types. Some of these types, such as ***Business Dynamics*** and ***Products Types***, are very similar to those identified in plant-based foods posts and thus, we focus on the remaining three categories: ***Regulations and Laws***, ***Research and Technology***, and ***Ethical Concerns*** (see Supporting Information S3 Appendix for more details). Although no space for discussion here, the temporal trend of these topics can be found in Supporting Information S4 Appendix.

From the topic modeling results for posts on plant-based vs. cell-based foods, we can both discover the overlaps and distinctions in public discourses. Common topics include product types, ethical concerns and business dynamics, highlighting same public concerns about AP foods. In comparison, significant differences are mainly reflected in the following aspects: firstly, posts of plant-based foods encompass a broader range of product types, including not only meat alternatives but also products like plant-based milk and even non-food items. Second, we seldom see posts focus on menus, recipes and baking instructions in the discussion of cell-based AP foods, which implies that cell-based foods are not widely integrated into daily life. Instead of menus and recipes, posts surrounding cell-based foods are more heavily oriented toward technology, regulatory processes, and legal frameworks, showing public uncertainty and skepticism about these emerging products. Furthermore, while there is a higher proportion of health and nutrition posts in plant-based foods discussion, discussions about the nutritional value and health effects of cell-based food are relatively limited. These divergences all suggest that public conversations about cell-based foods are still in the nascent stages, with issues of safety and consumption suitability yet to be widely addressed.

### Distribution of topics among different page categories

**Fig 6:**
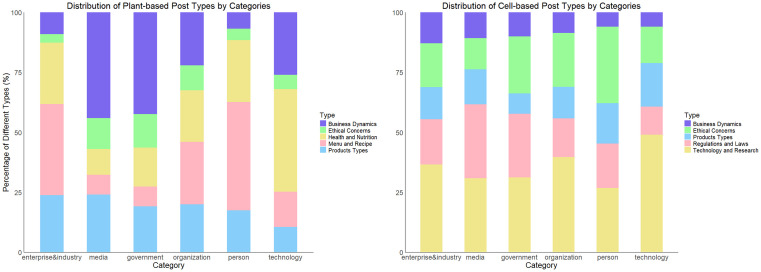
Distribution of Topics among Different Page Categories.

Then we further explore whether different page categories or actors emphasize different topics when talking about AP foods (see Fig 6). For plant-based foods, the person category primarily shares posts related to menus and recipes, while research institutions (technology) focus the most on the nutrition and health aspects. The highest proportion of posts on business dynamics and ethical concerns stem from media and government. Enterprises & industries tend to promote products and share menus and recipes through advertising, and organizations exhibit a relatively balanced focus across different themes. Regarding cell-based food, the scientific progress of lab-grown meat is the core agenda of research institutions (technology). The person and government pages show the greatest concern for ethical issues related to animal rights and environmental protection. Both media and government pay significant attention to the regulations and laws surrounding cell-based foods, while enterprises & industries are the category most focused on business dynamics.

In summary, the distribution of topics in different page categories aligns with our expectation, highlighting the finding that, in posts about plant-based foods, the media and government express more concern about business dynamics than the enterprises and industries, whereas the latter is more concerned with consumer outreach and product marketing. This suggests that the media and government are more attuned to the broader economic and structural impacts of the plant-based food industry. Furthermore, the heightened level of ethical concerns about cell-based foods among persons reinforce our earlier observation of growing public awareness and curiosity regarding the environmental and ethical implications of lab-grown meat production.

## Discussion

The continuous decline of public attention on plant-based AP foods may not be bad news. It shows that the publics might have accepted plant-based AP foods into their daily diets and considered the type of foods non-controversial. Taking into account the dominant positive sentiments and the everyday topics (e.g., products types and nutritional\health benefits) seen in plant-based discussions, we conclude that plant-based AP foods have been integrated into the publics’ daily life and the public acceptance of such foods can be further promoted without fundamental resistance. However, cell-based AP foods are still in their nascent stage in terms of public understanding. Despite heightened attention in the past decade, sentiments were mixed with a significant portion of negative views and frequently appearing topics still focused on basic ones such as whether the technology is mature enough to provide safe foods and how regulations should be in place to contain the risks. These concerns echo broader findings in the literature on public perception of emerging food technologies, where technological unfamiliarity often leads to skepticism and hesitancy [5]. We conclude that cell-based AP foods still face a considerable amount of public resistance and the controversy around the type of foods need to be carefully addressed.

The global distribution of public attention to AP foods is highly uneven. However, this finding may be related to both the global penetration rate of Facebook and disparities in levels of national AP foods development. According to Internet World Stats (2022), Facebook usage rates are highest in North America (82.3%) and Latin America (77.8%), with Europe, Oceania, and the Middle East following at the second level, while Asia and Africa remain below the global average at around 20%. This partially accounts for the lower levels of discussion about AP foods on Facebook in Asia and Africa. However, the disparity in Facebook discussions between North America and Latin America—despite their similarly high penetration rates—suggests that developed countries, with more advanced industries and greater scientific expertise, tend to generate higher public engagement with the posts, indicating a potential gap between developed and developing countries in public understanding of AP foods.

Finally, page categories analyses show that media, organizations, and enterprises & industries are the leading actors generating public discussions about AP foods. Government and technology pages lag behind in terms of both post volume and total engagement, a pattern consistent with previous studies highlighting the limited visibility and influence of governmental or scientific voices in online food discourse [25,28]. Although most page categories are generally positive about plant-based foods with more negativity associated with cell-based foods, it is notable that the category of persons (i.e., individuals) shows the most negative sentiments towards cell-based foods. If we take the person category as representative of ordinary people, we conclude that the general public is more concerned about cell-based AP foods’ ethical implications, technological development, and regulation, compared to plant-based AP foods. This paper concludes that for plant-based AP foods, public acceptance is higher and the challenge we face is to further increase its consumption. However, for cell-based AP foods, basic public consensus regarding its daily consumption is not established yet, as echoed in systematic reviews showing mixed public acceptance of cultured meat [11]. Among various social actors who can influence the publics, governmental and scientific discourses are not found to be very impactful yet.

## Conclusions

This study provides a systematic, longitudinal mapping of public discourses surrounding AP foods by analyzing English-language Facebook public pages from 2014 to 2024. Using computational methods, we tracked the evolving topics, sentiments, and key actors that have shaped the online discourse around plant-based and cell-based protein innovations. Our findings reveal a decline in public attention to plant-based AP foods since 2021, alongside an uneven but growing interest in cell-based alternatives. Our sentiment analysis shows that plant-based foods continue to receive predominantly positive responses, whereas cell-based foods elicit more mixed and polarized reactions. Thematically, discussions about plant-based proteins are broader and more practical, while cell-based discussions remain limited in scope. Importantly, industry, media, and organizations emerged as the dominant voices in this discourse. By leveraging large-scale social media data, this study complements existing research based on surveys and interviews and offers a data-rich perspective on the dynamics of public engagement with novel food technologies.

## Supporting information

S1 Fig Google Trends of Plant-based and Cell-based Novel Food over Years. (DOCX)

S2 Fig Number of Novel Foods Posts by Categories. (DOCX)

S1 Table Search Keywords. (DOCX)

S1 Appendix Data Pre-Processing. (DOCX)

S2 Appendix Categorization of Pages. (DOCX)

S3 Appendix Topic Modeling Results. (DOCX)

S4 Appendix Temporal Trend of Topics. (DOCX)
