# Supplementary figures and images for: Public discourses of alternative protein foods in Facebook public pages’ posts, 2014–2024
Source: PLoS One. 2025 Oct 17;20(10):e0333922. doi: 10.1371/journal.pone.0333922 (PMC12533839; doi:10.1371/journal.pone.0333922)

**S2 Figure. Number of Novel Foods Posts by Categories.**


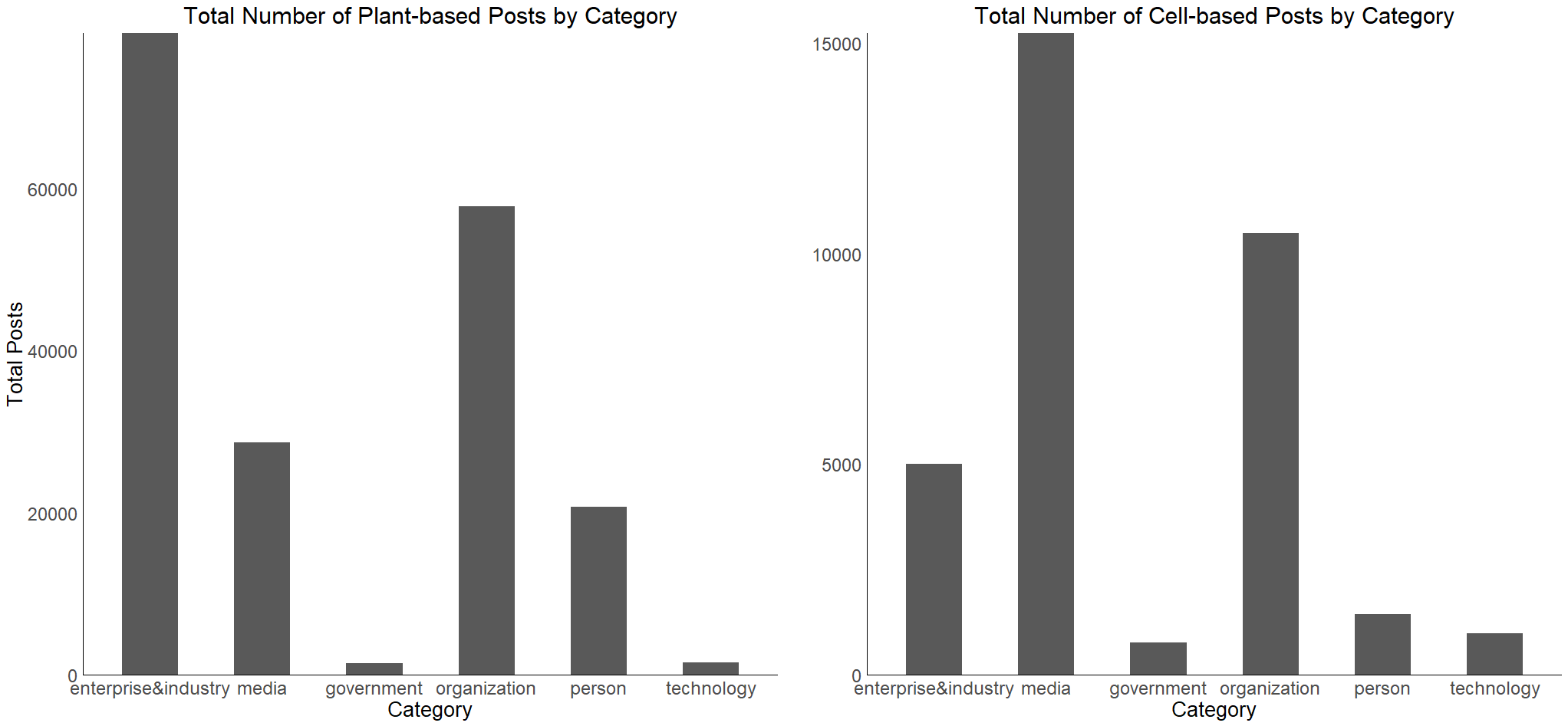

Supplement: S2 Fig — (DOCX) [file pone.0333922.s002.docx]
