# Supplementary material for: Public discourses of alternative protein foods in Facebook public pages’ posts, 2014–2024
Source: PLoS One. 2025 Oct 17;20(10):e0333922. doi: 10.1371/journal.pone.0333922 (PMC12533839; doi:10.1371/journal.pone.0333922)
Supplement: S1 Table — (DOCX) [file pone.0333922.s003.docx]

**S1 Table. Search Keywords.**

| **Type of Alternative Protein Foods** | **Keywords** |
| --- | --- |
| Plant-based | "Plant-based milk" OR "Plant-based dairy" OR "Plant-based egg" OR "Plant-based seafood" OR "Plant-based shellfish" OR "Plant-based fish" OR "Plant-based protein" OR "Plant-based meat" OR "Plant based milk" OR "Plant based dairy" OR "Plant based egg" OR "Plant based seafood" OR "Plant based shellfish" OR "Plant based fish" OR "Plant based protein" OR "Plant based meat" OR "vegan milk" OR "vegan dairy" OR "vegan egg" OR "vegan seafood" OR "vegan shellfish" OR "vegan fish" OR "vegan protein" OR "vegan meat" OR "Plant-based meats" OR "Plant based meats" OR "vegan meats" |
| Cell-based | ((("cell-based" OR "cell culture" OR "cell-culture" OR "cell cultured" OR "cell-cultured" OR "lab-grown" OR "lab grown") AND (meat OR meats OR seafood OR seafoods OR shellfish OR fish OR protein OR proteins)) NOT (virus OR cancer OR diamond OR mouse OR mice)) OR "cellular agriculture" OR "cultivated meat" OR "cultivated seafood" OR "cultivated shellfish" OR "cultivated fish" OR "cultivated protein" OR "cultured meat" OR "cultured seafood" OR "cultured shellfish" OR "cultured fish" OR "cultured protein" OR "synthetic meat" OR "synthetic seafood" OR "synthetic shellfish" OR "synthetic fish" OR "synthetic protein" |
