# Supplementary material for: Public discourses of alternative protein foods in Facebook public pages’ posts, 2014–2024
Source: PLoS One. 2025 Oct 17;20(10):e0333922. doi: 10.1371/journal.pone.0333922 (PMC12533839; doi:10.1371/journal.pone.0333922)
Supplement: S1 Appendix — (DOCX) [file pone.0333922.s004.docx]

**S1 Appendix. Data Pre-Processing.**

**Appendix A.1. Plant-Based Meat Message Cleaning Process**

**1. Preliminary Coding**

Two coders independently pre-coded a sample of 200 Facebook messages. The inter-coder reliability, assessed using Krippendorff’s alpha (α), was 0.966. Following the pre-coding phase, the coders reached a consensus on the criteria for coding messages relevant to plant-based meat. The coding criteria are listed in Table 1 below. Based on the established coding criteria, the two coders performed coding on an additional 800 Facebook messages.

Table A.1.1 Plant-Based Meat Coding Criteria

| Category | Criteria |
| --- | --- |
| **Messages Relevant to  Plant-based Meat** | 1. The Facebook text should contain at least one of the following keywords: "plant-based meat," "plant-based protein," "vegan milk," "vegan egg," "vegan dairy," or the name of a plant-based meat brand (e.g., Beyond).  2. Keywords related to plant-based proteins in Facebook text must appear in complete sentences. |
| **Messages Irrelevant to  Plant-based Meat** | 1. The Facebook text is not a complete sentence (e.g., it may consist solely of question marks or hashtags).  2. The Facebook text does not contain any of the specified keywords.  3. Even if the text contains the specified keywords, the following are excluded:  a. Keywords presented in HTML format.  b. Texts that describe a vegan diet without mentioning plant-based protein foods specifically, such as general descriptions of vegetarian habits. |

**2. Model Training**

We then use BERT model to classify the text as either relevant or irrelevant to plant-based meat. We firstly partitioned the dataset, allocating 80% for training and reserving the remaining 20% for validation. The training dataset was further divided into training and test subsets. We selected the model that demonstrated the best performance on the test dataset. The performance metrics for the chosen model are summarized in Table 2 below.

Table A.1.2 Model Performance on Test Dataset

| **Metric** | **Value** |
| --- | --- |
| Accuracy | 0.835 |
| Balanced Accuracy | 0.617 |
| Recall | 0.712 |
| F1 Score | 0.648 |

**3.Consistency Check**

To evaluate the agreement between the predictions generated by the BERT model and those provided by human coders, we analyzed the validation dataset. The computed Cohen’s Kappa value was found to be 0.759, as shown in Table 3.

| Table A.1.3 Cohen’s kappa | | | | | | | | | |
| --- | --- | --- | --- | --- | --- | --- | --- | --- | --- |
|  | | | | | | 95% CI | | | |
| Ratings | | kappa | | SE | | Lower | | Upper | |
| Average kappa |  | 0.759 |  |  |  |  |  |  |  |
| humanlabel - Predicted_Label |  | 0.759 |  | 0.048 |  | 0.664 |  | 0.853 |  |
|  | | | | | | | | | |
| *Note.*  200 subjects/items and 2 raters/measurements. Confidence intervals are asymptotic. | | | | | | | | | |

**Appendix A.2. Cell-Based Meat Message Cleaning Process**

**1. Preliminary Coding**

Two coders independently pre-coded a sample of 200 Facebook messages. The inter-coder reliability, assessed using Krippendorff’s alpha (α), was 0.879. Following the pre-coding phase, the coders reached a consensus on the criteria for coding messages relevant to cell-based meat. The coding criteria are listed in Table 1 below. Based on the established coding criteria, the two coders performed coding on an additional 800 Facebook messages.

Table A.2.1 Cell-Based Meat Coding Criteria

| Category | Criteria |
| --- | --- |
| **Messages Relevant to  Cell-based Meat** | 1. Keyword Presence: The Facebook text must include at least one of the following keywords or phrases: Synectic meat, Cell-based meat, Cultured meat, Man-made meat, Lab-grown meat (burger), Artificial meat, Varieties of meat (e.g., beef steak, seafood). 2. Complete Sentences: Keywords related to cell-based proteins must appear within the context of complete sentences. 3. A text that merely discusses cellular agriculture and cellular engineering cannot be counted as a text on cellular meat |
| **Messages Irrelevant to  Cell-based Meat** | 1. Incompleteness: The Facebook text must not be merely a string of punctuation (e.g., question marks or hashtags) without substantive content. 2. Keyword Absence: The text must not omit any of the specified keywords necessary for relevance. 3. HTML Format Exclusion: Even if the text contains specified keywords, it will be excluded if those keywords are presented in HTML format or any non-standard text format. |

**2. Model Training**

We then use BERT model to classify the text as either relevant or irrelevant to cell-based meat. We firstly partitioned the dataset, allocating 80% for training and reserving the remaining 20% for validation. The training dataset was further divided into training and test subsets. We selected the model that demonstrated the best performance on the test dataset. The performance metrics for the chosen model are summarized in Table 2 below.

Table A.2.2 Model Performance on Test Dataset

| **Metric** | **Value** |
| --- | --- |
| Accuracy | 0.925 |
| Precision | 0.812 |
| Recall | 0.878 |
| F1 Score | 0.844 |

**3.Consistency Check**

To evaluate the agreement between the predictions generated by the BERT model and those provided by human coders, we analyzed the validation dataset. The computed Cohen’s Kappa value was found to be 0.828, as shown in Table 3.

| Table A.2.3 Cohen’s kappa | | | | | | | | |  |  |
| --- | --- | --- | --- | --- | --- | --- | --- | --- | --- | --- |
|  | | | | | | 95% CI | | | | |
| Ratings | | kappa | | SE | | Lower | | Upper | | |
| Average kappa |  | 0.828 |  |  |  |  |  |  | |  |
| label - Predicted_Label |  | 0.828 |  | 0.059 |  | 0.713 |  | 0.943 | |  |
|  | | | | | | | | | | |
