# Supplementary material for: Public discourses of alternative protein foods in Facebook public pages’ posts, 2014–2024
Source: PLoS One. 2025 Oct 17;20(10):e0333922. doi: 10.1371/journal.pone.0333922 (PMC12533839; doi:10.1371/journal.pone.0333922)
Supplement: S2 Appendix — (DOCX) [file pone.0333922.s005.docx]

**S2 Appendix. Categorization of Pages.**

**Coding Criteria:**

1. For categories involving address entities, these are usually considered as "organization" categories, such as STREET, CAMPUS, and similarly, disciplines within categories are also considered a form of organization: NEUROSURGERY.

2. For entities with overlapping categories, the principle of function over form applies, i.e., science, media, enterprises, and government take precedence over organizations and individuals (except for artists like MUSICIAN, COMEDIAN, etc., who are classified under individuals).

3. In cases where science, media, enterprises, and government overlap, the classification standard is as follows:

(1) Enterprise and Media overlap:

a. If the entity clearly has media functions and products, even if it is also a service or industry organization, it should be classified under media: such as MEDIA AGENCY, MEDIA NEWS COMPANY, TOPIC BOOK STORE, VIDEO GAME STORE; otherwise, it should be considered an enterprise.

b. To ensure consistency, sports should all be classified under enterprise and industry: such as SPORTS AND RECREATION, TOPIC SPORTS RECREATION.

(2) Enterprise and Technology overlap:

a. If the entity clearly involves technology, medical, or engineering functions or products, even if it is also a service or industry organization, it should be classified under technology: such as ENGINEERING SERVICE, MEDICAL EQUIPMENT SUPPLIER, MEDICAL SERVICE, MEDICAL SUPPLIES, MEDICAL EQUIPMENT MANUFACTURER.

b. Market research institutions, individuals, and entities should be categorized under enterprise and industry: such as MARKET RESEARCH CONSULTANT.

(3) Enterprise and Government have no overlap.

(4) Media and Technology have no overlap.

(5) Media and Government have no overlap.

(6) Technology and Government have no overlap.

**Government and Administration (2%):**

1. Government institutions, personnel, and organizations: GOVERNMENT EMBASSY.

2. Administrative personnel, institutions, organizations: OFFICIAL CITY BOROUGH ARMY MILITARY.

3. Politicians: POLITICIAN.

**Technology (2%):**

1. Scientific personnel, institutions, organizations: SCIENTIST, SCIENCE ENGINEERING, SCIENCE SITE, PSYCHOLOGIST.

2. Specifically, medical personnel, institutions, organizations: MEDICAL SUPPLIES, MEDICAL SERVICE, MEDICAL LAB.

**Person (9%):**

1. Art creators: ARTIST, ACTOR, AUTHOR.

2. Professional and expert personnel: CHEF, COACH, GASTROENTEROLOGIST, ENDOCRINOLOGIST, NUTRITIONIST.

(Note: Some experts may belong to the business or technology sectors, but here they are classified under individuals, as we focus on the person’s views.)

3. Ordinary people and the public: PERSON, PERSONAL BLOG, FAN, BLOGGER.

**Media and Entertainment (7%):**

1. Electronic media, organizations, people: MOVIE, APP, TV, VIDEO GAME STORE, TOPIC PHOTOGRAPHER.

2. Traditional media, organizations, people: BOOK, NEWSPAPER, SONG, PODCAST.

3. Entertainment-related: FUN, ENTERTAINMENT, RECREATION.

**Enterprise & Industry (56%):**

1. Upstream agricultural enterprises, industries, organizations, people: AGRICULTURAL, AGRICULTURAL SERVICE, FARM.

2. Midstream food production and processing enterprises, organizations, people: FOOD BEVERAGE, FOOD COMPANY, FOOD STAND.

3. Downstream market industries, organizations, people: MARKETS, MARKETING, RESTAURANT, BAR, BREWERY.

4. Other industries and enterprises, organizations, people: WAXING SERVICE, WASTE MANAGEMENT, TENNIS, TEXTILES, REAL ESTATE DEVELOPER.

**Organization (24%):**

1. Addresses: STREET, CAMPUS, SCHOOL.

2. NGOs, Unions (with strong advocacy): NGO, CHARITY, COMMUNITY, CREDIT UNION, NON PROFIT.

3. Religious organizations: RELIGION, CHURCH, HINDU.

4. General organizations (describing a collective): CLUB, LOCAL, NEIGHBORHOOD, TEAM.

5. Calls for action, ideologies: ENVIRONMENTAL, HEALTH, ANIMAL WELFARE.
