# Supplementary material for: Public discourses of alternative protein foods in Facebook public pages’ posts, 2014–2024
Source: PLoS One. 2025 Oct 17;20(10):e0333922. doi: 10.1371/journal.pone.0333922 (PMC12533839; doi:10.1371/journal.pone.0333922)
Supplement: S3 Appendix — (DOCX) [file pone.0333922.s006.docx]

**S3 Appendix. Topic Modeling Results.**

1. **LDA Results of Plant-based Posts**


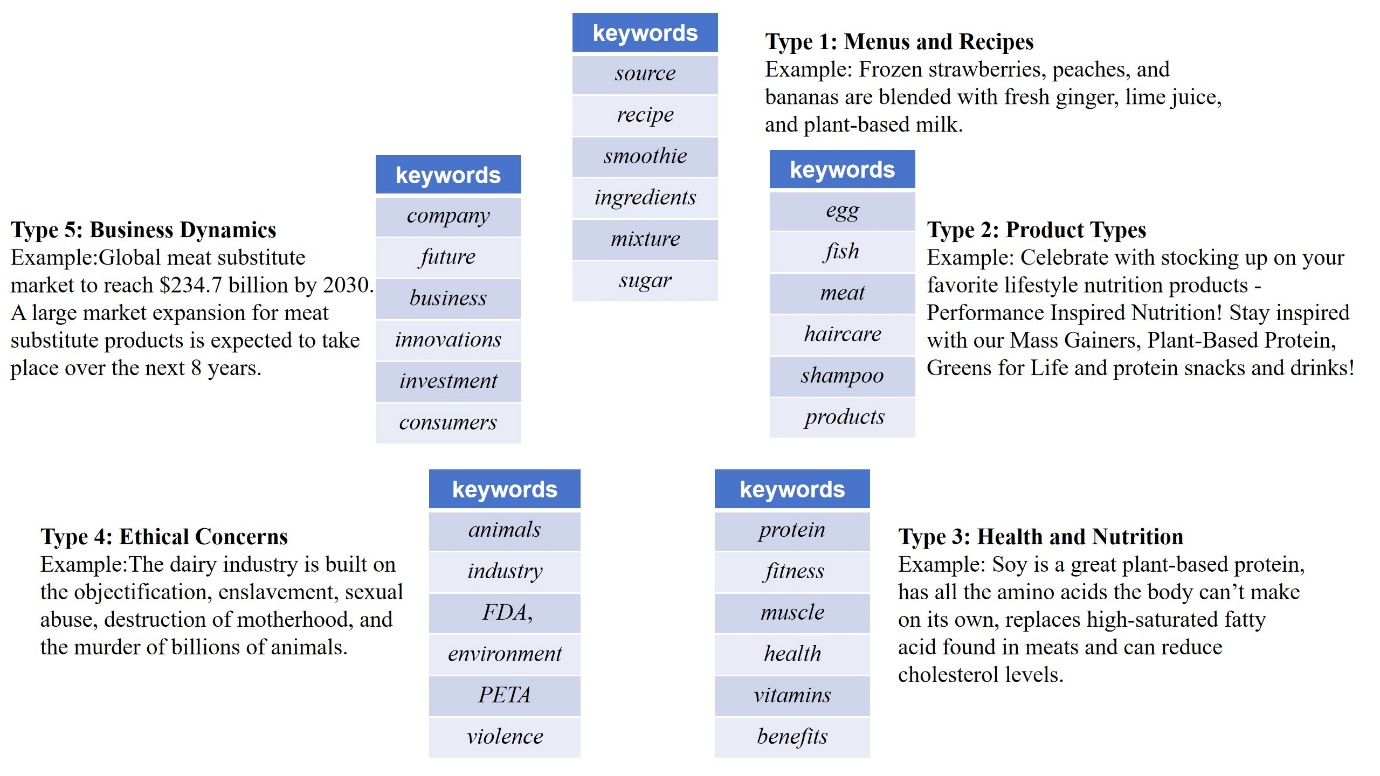


Figure C.1.1 LDA Results of Plant-based Food Posts

- ***Menus and Recipes***: Topics 1, 3, 5, and 6 fall under this type, which primarily includes posts featuring recipes or menu introductions for cooking with plant-based novel foods. High-frequency keywords such as *source*, *recipe*, *smoothie*, *sweet*, *sugar*, *ingredients*, *mixture*, *cup*, *add*, and *dish* highlight the focus on step-by-step cooking instructions, ingredient combinations, and meal preparation ideas. These posts often provide practical guidance on incorporating plant-based options into everyday meals.

*Example*: “*Frozen strawberries, peaches, and bananas are blended with fresh ginger, lime juice, and plant-based milk. The peachy-pink, digestion-friendly smoothie is served in bowls and topped with fresh kiwi, peaches, strawberries, coconut flakes, and buckwheat groats.*”

- ***Product Types:*** Topics 7 and 8 belong to this type, encompassing promotional posts from businesses advertising various plant-based products. High-frequency keywords such as *egg*, *fish*, *chicken*, *meat*, *burger*, *meal*, *taco*, *pizza*, *body*, *haircare*, *products*, and *shampoo* reflect the diverse range of items being promoted. These posts highlight not only alternative meat products, beverages, desserts, and alternative fish products but also extend to non-food items like skincare and haircare products, showcasing the expanding applications of plant-based innovations.

*Example: Spring is finally here! Celebrate with stocking up on your favorite lifestyle nutrition products - Performance Inspired Nutrition! Stay inspired with our Mass Gainers, Plant-Based Protein, Greens for Life and protein snacks and drinks!*

- ***Health and Nutrition***: Topics 4, 9, and 11 are grouped together, covering posts on the nutritional values and health benefits or potential risks of plant-based foods, with mixed opinions. High-frequency keywords such as *protein*, *muscle*, *fitness*, *benefits*, *disease*, *cardiovascular*, *mortality*, *vitamins*, *health*, *minerals*, and *energy* indicate a strong focus on the health-related aspects of plant-based foods. These posts explore plant-based foods’ role in enhancing physical fitness, preventing diseases, boosting energy levels, and delivering essential nutrients, while also addressing potential drawbacks.

*Example: Soy is a great plant-based protein, has all the amino acids the body can’t make on its own, replaces high-saturated fatty acid found in meats and can reduce cholesterol levels. However, there have been some studies that have questioned its benefit and have implied potential harm to the brain. After reviewing the data, it seems that the evidence of soy being beneficial by far outweighs any assumed risks.*

- ***Ethical Concerns***: Topic 2 falls into this type, mainly comprising discussions on animal welfare and sustainability. High-frequency keywords such as *animals*, *cows*, *industry*, *farms*, *giveaway*, *baby*, *FDA*, *environment*, *PETA*, *violence*, *cruel*, and *protest* underscore the ethical debates surrounding plant-based foods. These posts often highlight concerns about cruelty in traditional farming practices, the environmental impact of the livestock industry, and advocacy for sustainable and humane alternatives, frequently referencing organizations like PETA and related movements.

*Example: The dairy industry is built on the objectification, enslavement, sexual abuse, destruction of motherhood, and the murder of billions of animals. Dairy cows are forcibly impregnated over and over again to produce milk for the dairy industry. Calves born on the farms are separated from their mothers so that the milk that rightfully belongs to them could be sold to humans instead. Mother cows mourn the loss of their stolen babies for weeks.*

- ***Business Dynamics***: Topic 10 belongs to this type, including posts about the industry’s development prospects and market trends for plant-based novel foods. High-frequency keywords such as *company*, *future*, *industry*, *global*, *consumers*, *business*, *innovations*, *startup*, *CEO*, *investment*, and *frontier* highlight discussions on the evolving landscape of the plant-based food sector. These posts explore emerging business opportunities, innovative solutions, consumer trends, and investments shaping the future of this rapidly growing industry.

*Example: Global meat substitute market to reach $234.7 billion by 2030. A large market expansion for meat substitute products is expected to take place over the next 8 years. The global meat substitute market size is expected to reach USD 234.7 billion by 2030, according to a new report by Grand View Research. Furthermore, growing awareness of environmental and ethical issues has also accelerated market growth. Plant-based meat is a healthier alternative to traditional meat products.*

1. **LDA Results of Cell-based Posts**


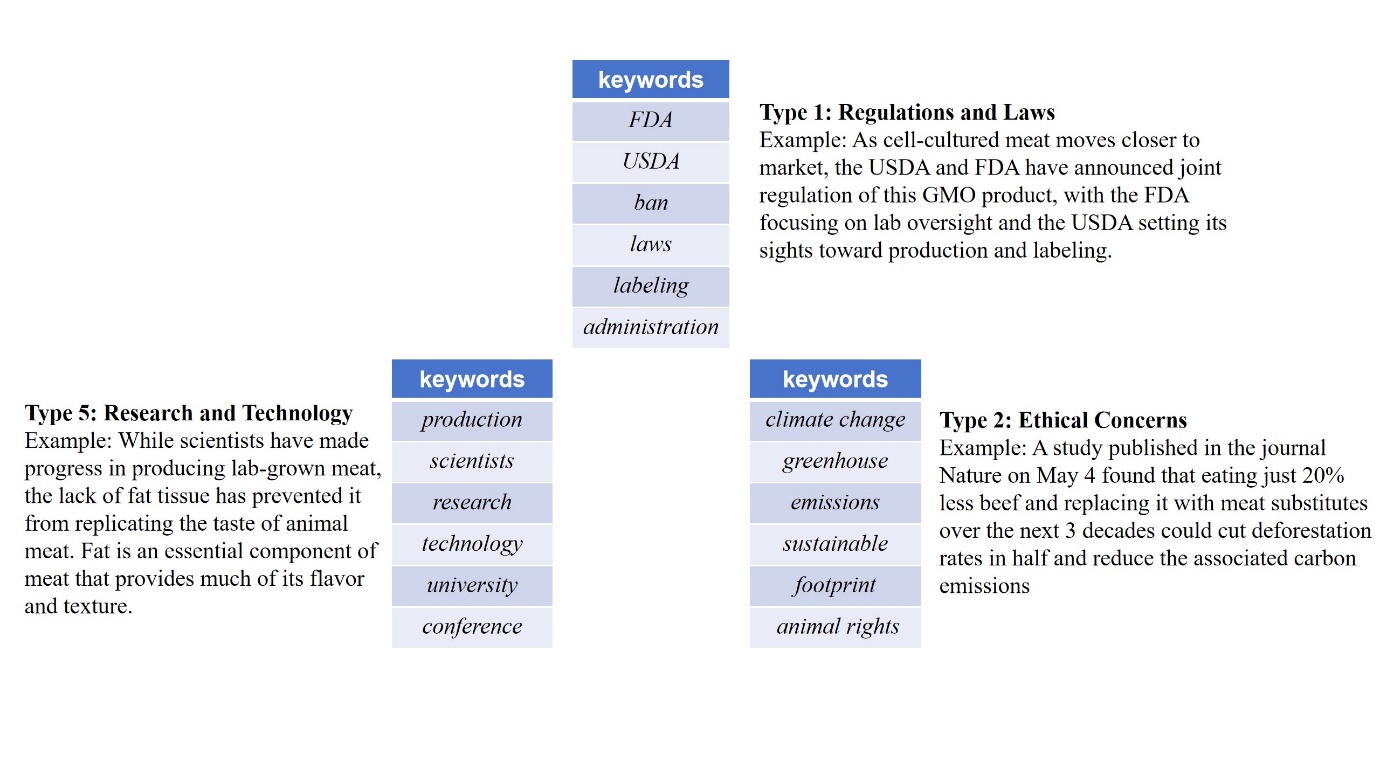
Figure C.2.1 LDA Results of Cell-based Food Posts

Some of these types, such as ***Business Dynamics*** and ***Product Types***, are very similar to those identified in plant-based foods posts and will not be discussed further here.

- ***Regulations and Laws*** (topic 1 and 10): Posts in this type often address issues such as the role of agencies like the FDA, debates over bans, laws, and approval processes, as well as concerns about labeling standards and administrative procedures. Keywords such as *FDA*, *USDA*, *ban*, *laws*, *approval*, *labeling*, *administration*, *government*, *regulatory*, and *allowing* are highly representative of the discourse within this category.

*Example: As cell-cultured meat moves closer to market, the USDA and FDA have announced joint regulation of this GMO product, with the FDA focusing on lab oversight and the USDA setting its sights toward production and labeling. FDA and USDA will jointly regulate cell-cultured meat.*

- ***Ethical Concerns*** (topic 2, 6 and 7): This type primarily consists of discussions on animal rights, human-animal relationships, resource scarcity, and environmental protection, particularly in the context of sustainable development. A notable difference from the plant-based foods posts in the ethical concerns is that, in the case of cell-based posts, people not only address animal rights but also emphasize issues such as food shortages, climate change, and broader sustainability concerns. There is a deeper reflection on the concept of veganism and its implications for sustainability. Keywords such as *climate change*, *greenhouse*, *emissions*, *sustainable*, *footprint*, *crisis*, *animal rights*, *veganism*, *pets*, and *human* are frequently associated with this discourse.

*Example: A study published in the journal Nature on May 4 found that eating just 20% less beef and replacing it with meat substitutes over the next 3 decades could cut deforestation rates in half and reduce the associated carbon emissions. Researchers at the Potsdam Institute for Climate Research in Germany found that if global beef consumption habits remain the same between 2020 and 2050, keeping up with growth in population, income, and livestock demand will require more land to be used for grazing and crop production.*

- ***Research and Technology*** (topic 3 and 5): This category is unique to the cell-based foods posts and focuses primarily on the scientific advancements, production processes, technological innovations, and academic contributions related to cell-based foods. Keywords such as *production*, *scientists*, *research*, *technology*, *innovation*, *university*, *conference*, *professor*, and *student* are commonly found in this discourse, highlighting the ongoing efforts in advancing the field and the role of academia in shaping the future of cell-based food.

*Example: Researchers have developed a method for producing lab-grown fat tissue that can be scaled up to make lab-grown meat more viable. While scientists have made progress in producing lab-grown meat, the lack of fat tissue has prevented it from replicating the taste of animal meat. Fat is an essential component of meat that provides much of its flavor and texture.*
