# Supplementary material for: Public discourses of alternative protein foods in Facebook public pages’ posts, 2014–2024
Source: PLoS One. 2025 Oct 17;20(10):e0333922. doi: 10.1371/journal.pone.0333922 (PMC12533839; doi:10.1371/journal.pone.0333922)
Supplement: S4 Appendix — (DOCX) [file pone.0333922.s007.docx]

**S4 Appendix. Temporal Trend of Topics.**

Here, we aim to find out the temporal trend of topics through the line plot of the proportions of each topic type over the past decades. As for the plant-based posts, ***Menus and Recipes*** consistently held a relatively high share, fluctuating around 30% of all posts, while it experienced a noticeable decline in 2019 and then return to its previous high levels in 2020. The most pronounced fluctuations occurred in the ***Health and Nutrition*** and ***Business Dynamics*** categories. Discussions on the nutritional and health value of plant-based foods declined steadily from 2014 to 2019 and then began to rebound thereafter, while discussions on Business Dynamics followed an opposite trend, which rose continuously from 2014 to 2019 and the upward trend reversed sharply after 2019. The categories with relatively smaller changes are ***Products Types*** and ***Ethical Concerns***. The former experienced a slight decline over the decade, stabilizing at around 20%, and the latter consistently ranked the lowest among all categories while it saw a significant increase during 2021–2022, temporarily surpassing 10% of all posts in 2022.


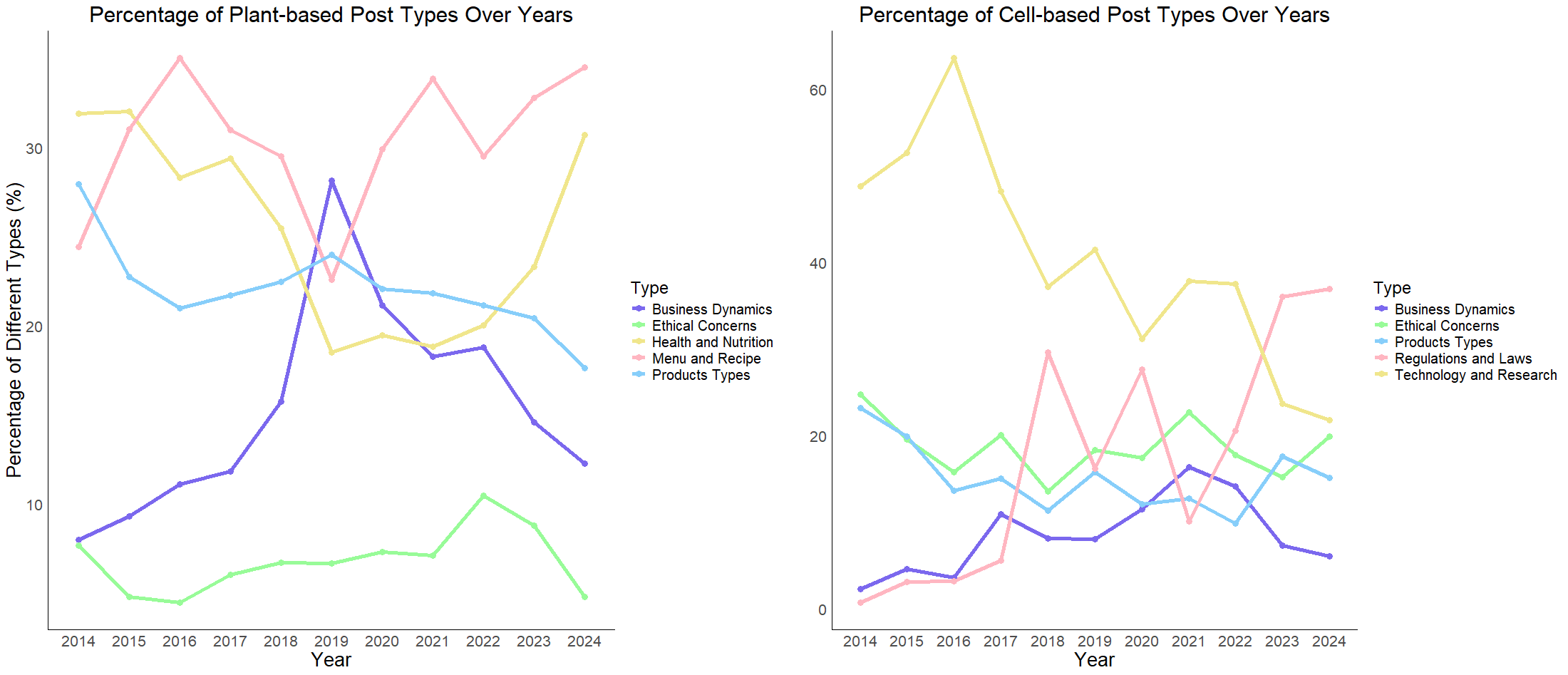


Figure D.1 Distribution of Different Thematic Types of Novel Food Posts over Years

As for the cell-based food posts, the topic of ***Technology and Research*** has consistently dominated the agenda, although its share among all posts has shown an overall notable declining trend. It peaked at over 60% in 2016 but decreased to just above 20% by 2024. In contrast to the downward trend, the theme showing a significant upward trajectory is ***Regulations and Laws***, which rose from a negligible proportion in 2014 to nearly 40% by 2024. The proportions of ***Ethical Concerns*** and ***Products Types*** have remained relatively stable, with the former consistently slightly higher than the latter, both hovering between 10% and 25%. In contrast, public discourses on ***Business Dynamics*** have persistently remained at very low levels, peaking around 15% in 2021 before falling back to a single-digit percentage by 2024.

The reason why public discourses on plant-based vs. cell-based foods exhibit clear differences in their thematic distribution may stem from differences in public familiarity with these novel foods, their levels of technological maturity, and the ethical and regulatory challenges they pose. The noticeable concentration on ***Menu and Recipes*** in discussion of plant-based foods demonstrates the public’s higher and growing familiarity with plant-based products, leading to an increase in practical applications like recipe sharing. Similarly, the precondition of ongoing debate about whether plant-based proteins can provide nutritional value equivalent to that of meat is that plant-based foods have entered the market on a large scale. In contrast, discourses on cell-based foods are predominantly centered on ***Technology and Research*** rather than concrete products, implying that public interest is driven by their curiosity towards emerging food technology. The continuous growth of ***Regulations and Laws*** type suggests a heightened public concentration in how these products will be governed and integrated into existing regulatory systems. In addition, there is a difference in the focus on Ethical Concerns about the two types of novel food, with people discussing ethical controversies with significantly more fervor in discussions of cell-based food. The unresolved questions about sustainability, including carbon emissions, resource use (e.g., water and energy), and the ethical implications of animal cell cultivation appear to drive more intense ethical debates around cell-based foods.
