# Supplementary material for: Public discourses of alternative protein foods in Facebook public pages’ posts, 2014–2024
Source: PLoS One. 2025 Oct 17;20(10):e0333922. doi: 10.1371/journal.pone.0333922 (PMC12533839; doi:10.1371/journal.pone.0333922)
Supplement: Attachment — Submitted filename: Comments.docx [file pone.0333922.s008.docx]

Dear Editor, **Dr. Issa Atoum**

Thank you for your request dated 25 June 2025 concerning the evaluation of manuscript number PONE-D-25-28924, entitled (Public Discourses of Alternative Protein Foods in Facebook Public Pages’ Posts, 2014-2024). Bellow my comments on this manuscript:

**General comment**: The manuscript is written in clear and good language; however, it lacks conclusions.

**Title**: Accurately reflects the content of the manuscript.

**Abstract**: The authors should include a concise conclusion at the end of the abstract.

**Keywords**: Should be arranged in alphabetical order.

**Introduction:** The authors should clearly state the objective of their study in detail at the end of the introduction.

**Materials and Methods**: The authors should clearly indicate the statistical analysis used for their study data.

**Results:** Clear and well presented.

**Discussion**: The authors should support their discussion of the study results with relevant scientific references and provide a more detailed analysis of the findings.

**References**: All references are listed without DOIs.

**Figures**: All figures are clear and informative, except for Figure 4, which requires higher resolution and clarity
