# Supplementary material for: Public discourses of alternative protein foods in Facebook public pages’ posts, 2014–2024
Source: PLoS One. 2025 Oct 17;20(10):e0333922. doi: 10.1371/journal.pone.0333922 (PMC12533839; doi:10.1371/journal.pone.0333922)
Supplement: Attachment — Submitted filename: Response to Reviewers.docx [file pone.0333922.s011.docx]

Response Letter

Dear Editor,

We sincerely thank the reviewers for their constructive comments and suggestions, which have significantly improved the quality of our manuscript. Below, we provide a detailed, point-by-point response to each comment and describe the corresponding revisions made in the manuscript. The response is divided to two parts: part 1 is to answer journal requirements and additional editor comments and part 2 to answer review comments.

Part 1.

1. Please ensure that your manuscript meets PLOS ONE’s style requirements, including those for file naming.

We revised the manuscript formatting and file names to fully comply with PLOS ONE’s style guidelines.

2. In your Methods section, please include additional information about your dataset and ensure that you have included a statement specifying whether the collection and analysis method complied with the terms and conditions for the source of the data.

We added detailed information about the dataset in the Methods section and included a statement confirming that the data collection and analysis complied with CrowdTangle’s terms of service.

3. Please include your full ethics statement in the ‘Methods’ section of your manuscript file. In your statement, please include the full name of the IRB or ethics committee who approved or waived your study, as well as whether or not you obtained informed written or verbal consent. If consent was waived for your study, please include this information as well.

We inserted a complete ethics statement in the Methods section, specifying the name of the ethics committee, the waiver of consent, and the justification for the waiver.

4. You indicated that data cannot be shared publicly because of CrowdTangle’s policy. However, PLOS ONE requires all data underlying findings to be made freely available unless restricted by ethical or legal constraints. Please revise accordingly.

We revised the Data Availability statement to clarify that the data are subject to third-party restrictions under CrowdTangle’s legal terms. NUS IRB imposed our compliance to data providers’ legal terms and CrowdTangle imposed the legal restrictions. We uploaded CrowdTangle’s terms of use in this resubmission.

5. Additional Comment: While there are clear edits in this version the issue of the data availability is still an open issue. Kindly share minimal data for review purposes which would not be placed in public repositories, where you can add in file inventory (for review only). The access to data should be fully detailed including how to grant access what special approval is required from.

We appreciate the reviewer’s comment. In accordance with CrowdTangle’s legal terms and the data usage policies of the National University of Singapore, we do not have the right to share the full dataset, but we are able to provide a randomly selected sample of the data for the journal’s review purposes. The detailed sample has been uploaded. Regarding the approval process, we do not have authorization from CrowdTangle to distribute the full dataset, and to our knowledge, the details of the approval procedure are not publicly available. Additionally, the official Facebook data platform (CrowdTangle) was closed in 2024.

In accordance with PLOS ONE’s third-party data policy, we note the following:

a) The manuscript describes the dataset and its source in detail.

b) We have included verification of our permission to use the data.

c) The data were obtained from the CrowdTangle platform, and other researchers could apply directly to CrowdTangle to access similar data before its closure.

6. When data cannot be publicly shared for ethical reasons, authors may make data available upon request or upload de-identified data as Supporting Information. Please update your Data Availability statement accordingly.

We confirmed the restrictions and clarified them in the revised Data Availability statement.

7. Please clarify the role of funders in the study. If the funders had no role, include the statement: “The funders had no role in study design, data collection and analysis, decision to publish, or preparation of the manuscript.”

We included the statement in the ‘Financial Disclosure’: “The funders had no role in study design, data collection and analysis, decision to publish, or preparation of the manuscript.”

8. The grant information in the ‘Funding Information’ and ‘Financial Disclosure’ sections does not match. Please ensure consistency.

We corrected and synchronized the grant numbers in both the ‘Funding Information’ and ‘Financial Disclosure’ sections.

9. Your abstract contains citations, which are not permitted. Please move all citations to the body of the text and maintain ascending numerical order.

We didn’t include citations in our abstract.

10. Figure 3 appears to contain copyrighted map/satellite imagery (e.g., from Google). PLOS ONE cannot publish such images unless permission for use under CC BY 4.0 is granted. Please either obtain permission or replace/remove the figure.

We confirm that Figure 3 was generated using the “map_data” function from an R package (map_data), which sources its base map from the CIA World DataBank. This data is in the public domain and complies with the journal’s copyright requirements. Proper citation has been included in the figure caption.

11. Please ensure that Figure 1 is cited in the manuscript text so that production can link the reader to the figure.

Thank you for pointing this out. We revised the manuscript to strengthen the linkage between Figure 1 and the main text by explicitly referencing and discussing the figure in the relevant section. This improves the coherence between the visual presentation and the narrative.

12. Please include captions for your Supporting Information files at the end of your manuscript and update the in-text citations accordingly.

We added captions for all Supporting Information files and updated the in-text citations to match.

Additional Editor Comments.

13. If the reviewer comments include a recommendation to cite specific previously published works, please review and evaluate these publications to determine whether they are relevant and should be cited. There is no requirement to cite these works unless the editor has indicated otherwise.

We note that the reviewer did not recommend citing any specific previously published works, and therefore no additional citations have been added in the revised manuscript.

14. While revising your submission, please upload your figure files to the Preflight Analysis and Conversion Engine (PACE) digital diagnostic tool, https://pacev2.apexcovantage.com/. PACE helps ensure that figures meet PLOS requirements.

We have uploaded all figure files to the PACE digital diagnostic tool and made necessary adjustments. All figures in the revised manuscript now comply with PLOS ONE’s requirements.

Part 2.

1. *General comment: The manuscript is written in clear and good language; however, it lacks conclusions.*

Thank you for your approval of our writing. Conclusions were added to the draft.

2. *Title: Accurately reflects the content of the manuscript.*

Thank you for your positive evaluation. No changes were required for the title.

3. *Abstract: The authors should include a concise conclusion at the end of the abstract.*

We appreciate this suggestion. A final sentence was added to the abstract that briefly concludes the main findings and their significance. (See Abstract, last sentence.)

4. *Keywords: Should be arranged in alphabetical order.*

The keywords were rearranged in alphabetical order as requested.

5. *Introduction: The authors should clearly state the objective of their study in detail at the end of the introduction.*

We revised the final paragraph of the Introduction to explicitly state the study’s objective in a clear and detailed manner. (See Introduction, last paragraph.)

6. *Materials and Methods: The authors should clearly indicate the statistical analysis used for their study data.*

The Methods section was revised to include a clear description of the statistical analyses employed in the study.

7. *Results: Clear and well presented.*

Thank you for the positive comment. No revisions were necessary in this section.

8. *Discussion: The authors should support their discussion of the study results with relevant scientific references and provide a more detailed analysis of the findings.*

As suggested, we substantially revised the Discussion section. We added multiple relevant scientific references (e.g., Onwezen et al., 2021; Ho et al., 2023) and elaborated on the interpretation of our findings in relation to the existing literature. (See revised Discussion section.)

9. *References: All references are listed without DOIs.*

We updated the reference list to include Digital Object Identifiers (DOIs) for all cited publications where available.

10. *Figures: All figures are clear and informative, except for Figure 4, which requires higher resolution and clarity.*

Figure 4 was replaced with a higher-resolution version to enhance clarity and ensure readability.
