# Supplementary material for: Public discourses of alternative protein foods in Facebook public pages’ posts, 2014–2024
Source: PLoS One. 2025 Oct 17;20(10):e0333922. doi: 10.1371/journal.pone.0333922 (PMC12533839; doi:10.1371/journal.pone.0333922)
Supplement: Peer review history

# Peer review: Decision Letter 0

**DOI:** 10.1371/journal.pone.0333922.r001

**Authors:** Issa Atoum

9 Jul 2025

PONE-D-25-28924Public Discourses of Alternative Protein Foods in Facebook Public Pages’ Posts, 2014-2024

PLOS ONE

Dear Dr. Zhang,

Thank you for submitting your manuscript to PLOS ONE. After careful consideration, we feel that it has merit but does not fully meet PLOS ONE’s publication criteria as it currently stands. Therefore, we invite you to submit a revised version of the manuscript that addresses the points raised during the review process.

Revisions are required before the manuscript can proceed to the next stage of the review process. These suggestions. Please revise the manuscript accordingly and prepare a point-by-point response document indicating how each comment has been addressed for the reviewer and the academic editor. Below is a consolidated summary of the required revisions of the methodology and paper structure.

The current Data Availability Statement does not comply with PLOS ONE’s Data Availability Policy (https://journals.plos.org/plosone/s/data-availability). The statement, “Data cannot be shared publicly because of CrowdTangle's policy. Data will be made available if the requestor can get special approval from CrowdTangle,” is insufficient. Authors must specify the reason for any access restrictions (such as licensing constraints), provide a public contact point or access mechanism (e.g., a URL to CrowdTangle’s data access process), and confirm that other researchers can access the data under the same conditions without requiring special privileges.

Please submit your revised manuscript by Aug 23 2025 11:59PM. If you will need more time than this to complete your revisions, please reply to this message or contact the journal office at plosone@plos.org . When you're ready to submit your revision, log on to https://www.editorialmanager.com/pone/ and select the 'Submissions Needing Revision' folder to locate your manuscript file.

Please include the following items when submitting your revised manuscript:

- A rebuttal letter that responds to each point raised by the academic editor and reviewer(s). You should upload this letter as a separate file labeled 'Response to Reviewers'.
- A marked-up copy of your manuscript that highlights changes made to the original version. You should upload this as a separate file labeled 'Revised Manuscript with Track Changes'.
- An unmarked version of your revised paper without tracked changes. You should upload this as a separate file labeled 'Manuscript'.

If you would like to make changes to your financial disclosure, please include your updated statement in your cover letter. Guidelines for resubmitting your figure files are available below the reviewer comments at the end of this letter.

If applicable, we recommend that you deposit your laboratory protocols in protocols.io to enhance the reproducibility of your results. Protocols.io assigns your protocol its own identifier (DOI) so that it can be cited independently in the future. For instructions see: https://journals.plos.org/plosone/s/submission-guidelines#loc-laboratory-protocols . Additionally, PLOS ONE offers an option for publishing peer-reviewed Lab Protocol articles, which describe protocols hosted on protocols.io. Read more information on sharing protocols at https://plos.org/protocols?utm_medium=editorial-email&utm_source=authorletters&utm_campaign=protocols .

We look forward to receiving your revised manuscript.

Kind regards,

Issa Atoum

Academic Editor

PLOS ONE

Journal Requirements:

When submitting your revision, we need you to address these additional requirements.

1. Please ensure that your manuscript meets PLOS ONE's style requirements, including those for file naming. The PLOS ONE style templates can be found at https://journals.plos.org/plosone/s/file?id=wjVg/PLOSOne_formatting_sample_main_body.pdf and https://journals.plos.org/plosone/s/file?id=ba62/PLOSOne_formatting_sample_title_authors_affiliations.pdf

2. In your Methods section, please include additional information about your dataset and ensure that you have included a statement specifying whether the collection and analysis method complied with the terms and conditions for the source of the data.

3. Please include your full ethics statement in the ‘Methods’ section of your manuscript file. In your statement, please include the full name of the IRB or ethics committee who approved or waived your study, as well as whether or not you obtained informed written or verbal consent. If consent was waived for your study, please include this information in your statement as well.

4. In the online submission form, you indicated that data cannot be shared publicly because of CrowdTangle's policy. Data will be made available if the requestor can get special approval from CrowdTangle.

All PLOS journals now require all data underlying the findings described in their manuscript to be freely available to other researchers, either 1. In a public repository, 2. Within the manuscript itself, or 3. Uploaded as supplementary information.

This policy applies to all data except where public deposition would breach compliance with the protocol approved by your research ethics board. If your data cannot be made publicly available for ethical or legal reasons (e.g., public availability would compromise patient privacy), please explain your reasons on resubmission and your exemption request will be escalated for approval.

5. We note that you have indicated that there are restrictions to data sharing for this study. For studies involving human research participant data or other sensitive data, we encourage authors to share de-identified or anonymized data. However, when data cannot be publicly shared for ethical reasons, we allow authors to make their data sets available upon request. For information on unacceptable data access restrictions, please see http://journals.plos.org/plosone/s/data-availability#loc-unacceptable-data-access-restrictions.

Before we proceed with your manuscript, please address the following prompts:

a) If there are ethical or legal restrictions on sharing a de-identified data set, please explain them in detail (e.g., data contain potentially identifying or sensitive patient information, data are owned by a third-party organization, etc.) and who has imposed them (e.g., a Research Ethics Committee or Institutional Review Board, etc.). Please also provide contact information for a data access committee, ethics committee, or other institutional body to which data requests may be sent.

b) If there are no restrictions, please upload the minimal anonymized data set necessary to replicate your study findings to a stable, public repository and provide us with the relevant URLs, DOIs, or accession numbers. Please see http://www.bmj.com/content/340/bmj.c181.long for guidelines on how to de-identify and prepare clinical data for publication. For a list of recommended repositories, please see https://journals.plos.org/plosone/s/recommended-repositories. You also have the option of uploading the data as Supporting Information files, but we would recommend depositing data directly to a data repository if possible.

Please update your Data Availability statement in the submission form accordingly.

6. Thank you for stating the following financial disclosure:

The project was supported by National University of Singapore through the ReImagine grant A-8002074-00-00 and A*Star through Singapore Food Story Theme 2 Grant A-8001371-00-00.

Please state what role the funders took in the study. If the funders had no role, please state: "The funders had no role in study design, data collection and analysis, decision to publish, or preparation of the manuscript."

If this statement is not correct you must amend it as needed.

Please include this amended Role of Funder statement in your cover letter; we will change the online submission form on your behalf.

7. We note that the grant information you provided in the ‘Funding Information’ and ‘Financial Disclosure’ sections do not match.

When you resubmit, please ensure that you provide the correct grant numbers for the awards you received for your study in the ‘Funding Information’ section.

8. Your abstract cannot contain citations. Please only include citations in the body text of the manuscript, and ensure that they remain in ascending numerical order on first mention.

9. We note that Figure 3 in your submission contain [map/satellite] images which may be copyrighted. All PLOS content is published under the Creative Commons Attribution License (CC BY 4.0), which means that the manuscript, images, and Supporting Information files will be freely available online, and any third party is permitted to access, download, copy, distribute, and use these materials in any way, even commercially, with proper attribution. For these reasons, we cannot publish previously copyrighted maps or satellite images created using proprietary data, such as Google software (Google Maps, Street View, and Earth). For more information, see our copyright guidelines: http://journals.plos.org/plosone/s/licenses-and-copyright.

We require you to either (1) present written permission from the copyright holder to publish these figures specifically under the CC BY 4.0 license, or (2) remove the figures from your submission:

a. You may seek permission from the original copyright holder of Figure 3 to publish the content specifically under the CC BY 4.0 license.

We recommend that you contact the original copyright holder with the Content Permission Form (http://journals.plos.org/plosone/s/file?id=7c09/content-permission-form.pdf) and the following text:

“I request permission for the open-access journal PLOS ONE to publish XXX under the Creative Commons Attribution License (CCAL) CC BY 4.0 (http://creativecommons.org/licenses/by/4.0/). Please be aware that this license allows unrestricted use and distribution, even commercially, by third parties. Please reply and provide explicit written permission to publish XXX under a CC BY license and complete the attached form.”

Please upload the completed Content Permission Form or other proof of granted permissions as an "Other" file with your submission.

In the figure caption of the copyrighted figure, please include the following text: “Reprinted from [ref] under a CC BY license, with permission from [name of publisher], original copyright [original copyright year].”

b. If you are unable to obtain permission from the original copyright holder to publish these figures under the CC BY 4.0 license or if the copyright holder’s requirements are incompatible with the CC BY 4.0 license, please either i) remove the figure or ii) supply a replacement figure that complies with the CC BY 4.0 license. Please check copyright information on all replacement figures and update the figure caption with source information. If applicable, please specify in the figure caption text when a figure is similar but not identical to the original image and is therefore for illustrative purposes only.

The following resources for replacing copyrighted map figures may be helpful:

USGS National Map Viewer (public domain): http://viewer.nationalmap.gov/viewer/

The Gateway to Astronaut Photography of Earth (public domain): http://eol.jsc.nasa.gov/sseop/clickmap/

Maps at the CIA (public domain): https://www.cia.gov/library/publications/the-world-factbook/index.html and https://www.cia.gov/library/publications/cia-maps-publications/index.html

NASA Earth Observatory (public domain): http://earthobservatory.nasa.gov/

Landsat: http://landsat.visibleearth.nasa.gov/

USGS EROS (Earth Resources Observatory and Science (EROS) Center) (public domain): http://eros.usgs.gov/#

Natural Earth (public domain): http://www.naturalearthdata.com/

10. Please ensure that you refer to Figure 1 in your text as, if accepted, production will need this reference to link the reader to the figure.

11. Please include captions for your Supporting Information files at the end of your manuscript, and update any in-text citations to match accordingly. Please see our Supporting Information guidelines for more information: http://journals.plos.org/plosone/s/supporting-information.

[Note: HTML markup is below. Please do not edit.]

Reviewers' comments:

Reviewer's Responses to Questions

**Comments to the Author**

1. Is the manuscript technically sound, and do the data support the conclusions?

The manuscript must describe a technically sound piece of scientific research with data that supports the conclusions. Experiments must have been conducted rigorously, with appropriate controls, replication, and sample sizes. The conclusions must be drawn appropriately based on the data presented. 

Reviewer #1: No

**********

2. Has the statistical analysis been performed appropriately and rigorously? 

Reviewer #1: N/A

**********

3. Have the authors made all data underlying the findings in their manuscript fully available?

The PLOS Data policy requires authors to make all data underlying the findings described in their manuscript fully available without restriction, with rare exception (please refer to the Data Availability Statement in the manuscript PDF file). The data should be provided as part of the manuscript or its supporting information, or deposited to a public repository. For example, in addition to summary statistics, the data points behind means, medians and variance measures should be available. If there are restrictions on publicly sharing data—e.g. participant privacy or use of data from a third party—those must be specified.

Reviewer #1: Yes

**********

4. Is the manuscript presented in an intelligible fashion and written in standard English?

PLOS ONE does not copyedit accepted manuscripts, so the language in submitted articles must be clear, correct, and unambiguous. Any typographical or grammatical errors should be corrected at revision, so please note any specific errors here.

Reviewer #1: Yes

**********

5. Review Comments to the Author

Please use the space provided to explain your answers to the questions above. You may also include additional comments for the author, including concerns about dual publication, research ethics, or publication ethics. (Please upload your review as an attachment if it exceeds 20,000 characters)

Reviewer #1: General comment: The manuscript is written in clear and good language; however, it lacks conclusions.

Title: Accurately reflects the content of the manuscript.

Abstract: The authors should include a concise conclusion at the end of the abstract.

Keywords: Should be arranged in alphabetical order.

Introduction: The authors should clearly state the objective of their study in detail at the end of the introduction.

Materials and Methods: The authors should clearly indicate the statistical analysis used for their study data.

Results: Clear and well presented.

Discussion: The authors should support their discussion of the study results with relevant scientific references and provide a more detailed analysis of the findings.

References: All references are listed without DOIs.

Figures: All figures are clear and informative, except for Figure 4, which requires higher resolution and clarity

**********

6. PLOS authors have the option to publish the peer review history of their article (what does this mean? ). If published, this will include your full peer review and any attached files.

If you choose “no”, your identity will remain anonymous but your review may still be made public.

**Do you want your identity to be public for this peer review?** For information about this choice, including consent withdrawal, please see our Privacy Policy .

Reviewer #1: No

**********

[NOTE: If reviewer comments were submitted as an attachment file, they will be attached to this email and accessible via the submission site. Please log into your account, locate the manuscript record, and check for the action link "View Attachments". If this link does not appear, there are no attachment files.]

While revising your submission, please upload your figure files to the Preflight Analysis and Conversion Engine (PACE) digital diagnostic tool, https://pacev2.apexcovantage.com/ . PACE helps ensure that figures meet PLOS requirements. To use PACE, you must first register as a user. Registration is free. Then, login and navigate to the UPLOAD tab, where you will find detailed instructions on how to use the tool. If you encounter any issues or have any questions when using PACE, please email PLOS at figures@plos.org . Please note that Supporting Information files do not need this step.

Attachment Submitted filename: Comments.docx

# Author comment: Author response to Decision Letter 1

**DOI:** 10.1371/journal.pone.0333922.r002

20 Aug 2025

see attached response letter.

Attachment Submitted filename: Plos One Response to Comments-0816.docx

# Editor report: Decision Letter 1

**DOI:** 10.1371/journal.pone.0333922.r003

**Authors:** Issa Atoum

30 Aug 2025

PONE-D-25-28924R1Public Discourses of Alternative Protein Foods in Facebook Public Pages’ Posts, 2014-2024PLOS ONE

Dear Dr. Wang,

Thank you for submitting your manuscript to PLOS ONE. After careful consideration, we feel that it has merit but does not fully meet PLOS ONE’s publication criteria as it currently stands. Therefore, we invite you to submit a revised version of the manuscript that addresses the points raised during the review process.

Please submit your revised manuscript by Oct 14 2025 11:59PM. If you will need more time than this to complete your revisions, please reply to this message or contact the journal office at plosone@plos.org . When you're ready to submit your revision, log on to https://www.editorialmanager.com/pone/ and select the 'Submissions Needing Revision' folder to locate your manuscript file.

Please include the following items when submitting your revised manuscript:

- A rebuttal letter that responds to each point raised by the academic editor and reviewer(s). You should upload this letter as a separate file labeled 'Response to Reviewers'.
- A marked-up copy of your manuscript that highlights changes made to the original version. You should upload this as a separate file labeled 'Revised Manuscript with Track Changes'.
- An unmarked version of your revised paper without tracked changes. You should upload this as a separate file labeled 'Manuscript'.

If you would like to make changes to your financial disclosure, please include your updated statement in your cover letter. Guidelines for resubmitting your figure files are available below the reviewer comments at the end of this letter.

If applicable, we recommend that you deposit your laboratory protocols in protocols.io to enhance the reproducibility of your results. Protocols.io assigns your protocol its own identifier (DOI) so that it can be cited independently in the future. For instructions see: https://journals.plos.org/plosone/s/submission-guidelines#loc-laboratory-protocols . Additionally, PLOS ONE offers an option for publishing peer-reviewed Lab Protocol articles, which describe protocols hosted on protocols.io. Read more information on sharing protocols at https://plos.org/protocols?utm_medium=editorial-email&utm_source=authorletters&utm_campaign=protocols .

We look forward to receiving your revised manuscript.

Kind regards,

Issa Atoum

Academic Editor

PLOS ONE

Journal Requirements:

1. If the reviewer comments include a recommendation to cite specific previously published works, please review and evaluate these publications to determine whether they are relevant and should be cited. There is no requirement to cite these works unless the editor has indicated otherwise. 

Additional Editor Comments:

While their are clear edits in this version the issue of the data availability is still an open issue. Kindly share minimal data for review purposes which would not be placed in public repositories, where you can add in file inventory (for review only). The access to data should be fully detailed including how to grant access what special approval is required from CrowdTangle, kindly detail this approval requirements. The current Data Availability Statement does not comply with PLOS ONE’s Data Availability Policy (https://journals.plos.org/plosone/s/data-availability).

[Note: HTML markup is below. Please do not edit.]

[NOTE: If reviewer comments were submitted as an attachment file, they will be attached to this email and accessible via the submission site. Please log into your account, locate the manuscript record, and check for the action link "View Attachments". If this link does not appear, there are no attachment files.]

While revising your submission, please upload your figure files to the Preflight Analysis and Conversion Engine (PACE) digital diagnostic tool, https://pacev2.apexcovantage.com/ . PACE helps ensure that figures meet PLOS requirements. To use PACE, you must first register as a user. Registration is free. Then, login and navigate to the UPLOAD tab, where you will find detailed instructions on how to use the tool. If you encounter any issues or have any questions when using PACE, please email PLOS at figures@plos.org . Please note that Supporting Information files do not need this step.

# Author comment: Author response to Decision Letter 2

**DOI:** 10.1371/journal.pone.0333922.r004

18 Sep 2025

Response Letter

Dear Editor,

We sincerely thank the reviewers for their constructive comments and suggestions, which have significantly improved the quality of our manuscript. Below, we provide a detailed, point-by-point response to each comment and describe the corresponding revisions made in the manuscript. The response is divided to two parts: part 1 is to answer journal requirements and additional editor comments and part 2 to answer review comments.

Part 1.

1. Please ensure that your manuscript meets PLOS ONE’s style requirements, including those for file naming.

We revised the manuscript formatting and file names to fully comply with PLOS ONE’s style guidelines.

2. In your Methods section, please include additional information about your dataset and ensure that you have included a statement specifying whether the collection and analysis method complied with the terms and conditions for the source of the data.

We added detailed information about the dataset in the Methods section and included a statement confirming that the data collection and analysis complied with CrowdTangle’s terms of service.

3. Please include your full ethics statement in the ‘Methods’ section of your manuscript file. In your statement, please include the full name of the IRB or ethics committee who approved or waived your study, as well as whether or not you obtained informed written or verbal consent. If consent was waived for your study, please include this information as well.

We inserted a complete ethics statement in the Methods section, specifying the name of the ethics committee, the waiver of consent, and the justification for the waiver.

4. You indicated that data cannot be shared publicly because of CrowdTangle’s policy. However, PLOS ONE requires all data underlying findings to be made freely available unless restricted by ethical or legal constraints. Please revise accordingly.

We revised the Data Availability statement to clarify that the data are subject to third-party restrictions under CrowdTangle’s legal terms. NUS IRB imposed our compliance to data providers’ legal terms and CrowdTangle imposed the legal restrictions. We uploaded CrowdTangle’s terms of use in this resubmission.

5. Additional Comment: While there are clear edits in this version the issue of the data availability is still an open issue. Kindly share minimal data for review purposes which would not be placed in public repositories, where you can add in file inventory (for review only). The access to data should be fully detailed including how to grant access what special approval is required from.

We appreciate the reviewer’s comment. In accordance with CrowdTangle’s legal terms and the data usage policies of the National University of Singapore, we do not have the right to share the full dataset, but we are able to provide a randomly selected sample of the data for the journal’s review purposes. The detailed sample has been uploaded. Regarding the approval process, we do not have authorization from CrowdTangle to distribute the full dataset, and to our knowledge, the details of the approval procedure are not publicly available. Additionally, the official Facebook data platform (CrowdTangle) was closed in 2024.

In accordance with PLOS ONE’s third-party data policy, we note the following:

a) The manuscript describes the dataset and its source in detail.

b) We have included verification of our permission to use the data.

c) The data were obtained from the CrowdTangle platform, and other researchers could apply directly to CrowdTangle to access similar data before its closure.

6. When data cannot be publicly shared for ethical reasons, authors may make data available upon request or upload de-identified data as Supporting Information. Please update your Data Availability statement accordingly.

We confirmed the restrictions and clarified them in the revised Data Availability statement.

7. Please clarify the role of funders in the study. If the funders had no role, include the statement: “The funders had no role in study design, data collection and analysis, decision to publish, or preparation of the manuscript.”

We included the statement in the ‘Financial Disclosure’: “The funders had no role in study design, data collection and analysis, decision to publish, or preparation of the manuscript.”

8. The grant information in the ‘Funding Information’ and ‘Financial Disclosure’ sections does not match. Please ensure consistency.

We corrected and synchronized the grant numbers in both the ‘Funding Information’ and ‘Financial Disclosure’ sections.

9. Your abstract contains citations, which are not permitted. Please move all citations to the body of the text and maintain ascending numerical order.

We didn’t include citations in our abstract.

10. Figure 3 appears to contain copyrighted map/satellite imagery (e.g., from Google). PLOS ONE cannot publish such images unless permission for use under CC BY 4.0 is granted. Please either obtain permission or replace/remove the figure.

We confirm that Figure 3 was generated using the “map_data” function from an R package (map_data), which sources its base map from the CIA World DataBank. This data is in the public domain and complies with the journal’s copyright requirements. Proper citation has been included in the figure caption.

11. Please ensure that Figure 1 is cited in the manuscript text so that production can link the reader to the figure.

Thank you for pointing this out. We revised the manuscript to strengthen the linkage between Figure 1 and the main text by explicitly referencing and discussing the figure in the relevant section. This improves the coherence between the visual presentation and the narrative.

12. Please include captions for your Supporting Information files at the end of your manuscript and update the in-text citations accordingly.

We added captions for all Supporting Information files and updated the in-text citations to match.

Additional Editor Comments.

13. If the reviewer comments include a recommendation to cite specific previously published works, please review and evaluate these publications to determine whether they are relevant and should be cited. There is no requirement to cite these works unless the editor has indicated otherwise.

We note that the reviewer did not recommend citing any specific previously published works, and therefore no additional citations have been added in the revised manuscript.

14. While revising your submission, please upload your figure files to the Preflight Analysis and Conversion Engine (PACE) digital diagnostic tool, https://pacev2.apexcovantage.com/. PACE helps ensure that figures meet PLOS requirements.

We have uploaded all figure files to the PACE digital diagnostic tool and made necessary adjustments. All figures in the revised manuscript now comply with PLOS ONE’s requirements.

Part 2.

1. General comment: The manuscript is written in clear and good language; however, it lacks conclusions.

Thank you for your approval of our writing. Conclusions were added to the draft.

2. Title: Accurately reflects the content of the manuscript.

Thank you for your positive evaluation. No changes were required for the title.

3. Abstract: The authors should include a concise conclusion at the end of the abstract.

We appreciate this suggestion. A final sentence was added to the abstract that briefly concludes the main findings and their significance. (See Abstract, last sentence.)

4. Keywords: Should be arranged in alphabetical order.

The keywords were rearranged in alphabetical order as requested.

5. Introduction: The authors should clearly state the objective of their study in detail at the end of the introduction.

We revised the final paragraph of the Introduction to explicitly state the study’s objective in a clear and detailed manner. (See Introduction, last paragraph.)

6. Materials and Methods: The authors should clearly indicate the statistical analysis used for their study data.

The Methods section was revised to include a clear description of the statistical analyses employed in the study.

7. Results: Clear and well presented.

Thank you for the positive comment. No revisions were necessary in this section.

8. Discussion: The authors should support their discussion of the study results with relevant scientific references and provide a more detailed analysis of the findings.

As suggested, we substantially revised the Discussion section. We added multiple relevant scientific references (e.g., Onwezen et al., 2021; Ho et al., 2023) and elaborated on the interpretation of our findings in relation to the existing literature. (See revised Discussion section.)

9. References: All references are listed without DOIs.

We updated the reference list to include Digital Object Identifiers (DOIs) for all cited publications where available.

10. Figures: All figures are clear and informative, except for Figure 4, which requires higher resolution and clarity.

Figure 4 was replaced with a higher-resolution version to enhance clarity and ensure readability.

Sincerely,

Bingyan Wang

Department of Political Science, Tsinghua University

Attachment Submitted filename: Response to Reviewers.docx

# Editor report: Decision Letter 2

**DOI:** 10.1371/journal.pone.0333922.r005

**Authors:** Issa Atoum

22 Sep 2025

Public Discourses of Alternative Protein Foods in Facebook Public Pages’Posts, 2014–2024

PONE-D-25-28924R2

Dear Dr. Wang,

We’re pleased to inform you that your manuscript has been judged scientifically suitable for publication and will be formally accepted for publication once it meets all outstanding technical requirements.

Within one week, you’ll receive an e-mail detailing the required amendments. When these have been addressed, you’ll receive a formal acceptance letter and your manuscript will be scheduled for publication.

An invoice will be generated when your article is formally accepted. Please note, if your institution has a publishing partnership with PLOS and your article meets the relevant criteria, all or part of your publication costs will be covered. Please make sure your user information is up-to-date by logging into Editorial Manager at Editorial Manager®  and clicking the ‘Update My Information' link at the top of the page. For questions related to billing, please contact billing support .

If your institution or institutions have a press office, please notify them about your upcoming paper to help maximize its impact. If they’ll be preparing press materials, please inform our press team as soon as possible -- no later than 48 hours after receiving the formal acceptance. Your manuscript will remain under strict press embargo until 2 pm Eastern Time on the date of publication. For more information, please contact onepress@plos.org.

Kind regards,

Issa Atoum

Academic Editor

PLOS ONE

Additional Editor Comments (optional):

Reviewers' comments:

# Editor report: Acceptance letter

**DOI:** 10.1371/journal.pone.0333922.r006

**Authors:** Issa Atoum

PONE-D-25-28924R2

PLOS ONE

Dear Dr. Wang,

I'm pleased to inform you that your manuscript has been deemed suitable for publication in PLOS ONE. Congratulations! Your manuscript is now being handed over to our production team.

At this stage, our production department will prepare your paper for publication. This includes ensuring the following:

* All references, tables, and figures are properly cited

* All relevant supporting information is included in the manuscript submission,

* There are no issues that prevent the paper from being properly typeset

You will receive further instructions from the production team, including instructions on how to review your proof when it is ready. Please keep in mind that we are working through a large volume of accepted articles, so please give us a few days to review your paper and let you know the next and final steps.

Lastly, if your institution or institutions have a press office, please let them know about your upcoming paper now to help maximize its impact. If they'll be preparing press materials, please inform our press team within the next 48 hours. Your manuscript will remain under strict press embargo until 2 pm Eastern Time on the date of publication. For more information, please contact onepress@plos.org.

You will receive an invoice from PLOS for your publication fee after your manuscript has reached the completed accept phase. If you receive an email requesting payment before acceptance or for any other service, this may be a phishing scheme. Learn how to identify phishing emails and protect your accounts at https://explore.plos.org/phishing.

If we can help with anything else, please email us at customercare@plos.org.

Thank you for submitting your work to PLOS ONE and supporting open access.

Kind regards,

PLOS ONE Editorial Office Staff

on behalf of

Dr. Issa Atoum

Academic Editor

PLOS ONE
